# The function of prohibitins in mitochondria and the clinical potentials

**DOI:** 10.1186/s12935-022-02765-x

**Published:** 2022-11-08

**Authors:** Linda Oyang, Jian Li, Xianjie Jiang, Jinguan Lin, Longzheng Xia, Lixia Yang, Shiming Tan, Nayiyuan Wu, Yaqian Han, Yiqing Yang, Xia Luo, Jinyun Li, Qianjin Liao, Yingrui Shi, Yujuan Zhou

**Affiliations:** 1 Hunan Key Laboratory of Cancer Metabolism, Hunan Cancer Hospital and the Affiliated Cancer Hospital of Xiangya School of Medicine, Central South University, Changsha, 410013 Hunan China; 2 Department of Head and Neck Surgery, Hunan Cancer Hospital and the Affiliated Cancer Hospital of Xiangya School of Medicine, Central South University, Changsha, 410013 Hunan China; 3 Hunan Key Laboratory of Translational Radiation Oncology, 283 Tongzipo Road, Changsha, 410013 Hunan China

**Keywords:** Prohibitin, Mitochondria, Mitophagy, Mitochondrial dynamics, Mitochondrial biogenesis and quality control, Cancer, Mitochondria disease

## Abstract

Prohibitins (PHBs) are a class of highly evolutionarily conserved proteins that widely distribute in prokaryotes and eukaryotes. PHBs function in cell growth and proliferation or differentiation, regulating metabolism and signaling pathways. PHBs have different subcellular localization in eukaryotes, but they are mainly located in mitochondria. In the mitochondria, PHBs stabilize the structure of the mitochondrial membrane and regulate mitochondrial autophagy, mitochondrial dynamics, mitochondrial biogenesis and quality control, and mitochondrial unfolded protein response. PHBs has shown to be associated with many diseases, such as mitochondria diseases, cancers, infectious diseases, and so on. Some molecule targets of PHBs can interfere with the occurrence and development of diseases. Therefore, this review clarifies the functions of PHBs in mitochondria, and provides a summary of the potential values in clinics.

## Background

Prohibitins (PHBs) in mammals are divided into PHB1 and PHB2. PHB1, also known as anti-proliferative protein or BAP32, is named because of the scientific hypothesis when it was first discovered. Later, it was found that its function, such as growth cycles, pathology, and organelles, was different in various cellular compartments [1–3]. PHB2, also known as BAP37, is a protein that inhibits ER (estrogen receptor) by binding with ERα [4]. The function of its mitochondria has attracted attention in recent years. The molecular mechanisms by which the PHBs function in mitochondria and the potential clinical practice are reviewed and discussed herein. Please see Table 1 for the functions and mechanisms of PHBs in different cells/diseases.

**Table 1:** The functions and mechanisms of PHBs in different cells/diseases

Cells/diseases		Functions/mechanisms	References
Cells	Melanocytes	Rab27a-PHB-MLPH complex control melanosome	[14]
	Intestinal mucosal epithelial cells	The receptor of some viruses	[15–18]
	Hepatocytes	Mediate the virus to enter	[19]
	Renal cells	Induce hydrolytic inactivation of OPA1 protein; decrease mitochondrial dynamics	[45]
	Preadipocytes	Mitochondrial dysfunction and down-regulation of adipogenesis	[48, 49]
	Heart cells	Induce cardiac fatty acid metabolism disorders	[50]
Virus	EV-A71	Reduce virus replication and the incidence of critical illness	[86]
	COVID-19	Mediate the host immune evasion response	[87]
	New coronary pneumonia	Grant the ability of host immune evasion and virus replication	[88]
Diseases	AD	Regulate the mitochondrial oxidative stress response, reduce cognitive deficits	[31]
		Protect dopaminergic neurons against mitochondrial ROS and cytotoxicity	[58]
	HCV	PHB-CRAF interaction	[19]
	Triple-negative breast cancer	Dissociate from DRP1; disrupt the mitochondrial membrane potential and triggering the apoptotic cascade	[42]
	Gynecologic cancer	Prevent cisplatin-induced mitochondrial division and Oma1-mediated cleavage as well as changes in OPA1 processing	[46]
	Nasopharyngeal carcinoma	Counteract TRIM21-mediated ubiquitination to inhibit the NF-κB activity	[71]
	Hepatocellular carcinoma	Down-regulate the regulatory proteins in the G0/G1 phase	[72]
	Gastric cancer	MAPK signaling pathway decreases the ubiquitination level of PHB1	[73, 74]
	Colorectal carcinoma cells	Inhibit Wnt/beta-catenin signaling	[78]

## PHBs structure

PHB gene is located at q21 of chromosome 17 in human. The relative molecular weights of PHB1 and PHB2 are 32 kDa and 34 kDa, respectively, both of which have an N-terminal transmembrane domain and C-terminal helical domain. The N-terminus helps PHBs to locate the mitochondrial intima of the cell. Because of its estrogen receptor binding domain, the subcellular localization of PHB2 is more specific than PHB1. Between the N-terminal and C-termini, there is a PHB domain, also known as the SPFH domain [5]. PHB1 and PHB2 play a role as biomolecules alone but can also combine to form complexes to play a biological role or combine with other biomolecules to play a unique role. PHBs molecules in the form of monomers are easily to be degraded. Therefore, PHBs often function in the form of complexes. In mammalian mitochondria, it is mainly expressed in the form of PHB1/PHB2 heterodimer, PHB2-HAX1-ANT2-VDAC2 complex, PHB1-ANX2-CD36 complex [6], and DNAJC19-PHB2 complex [7]. In the cytoplasm, the PHB1 complexes can also form homotetramers to play the role of the cytoskeleton signaling pathway. In the nucleus, PHB1 acts as PHB1-HIRAC complex [8], PHB1-Brg1-Brm complex [9], or PHB1-STAT3-SH2D4A complex [10] and can directly bind to p53, E2F, Rb, c-Myc and other genes to regulate gene transcription and translation [6, 11, 12]

## Expression and functions of PHBs

**Fig. 1:**
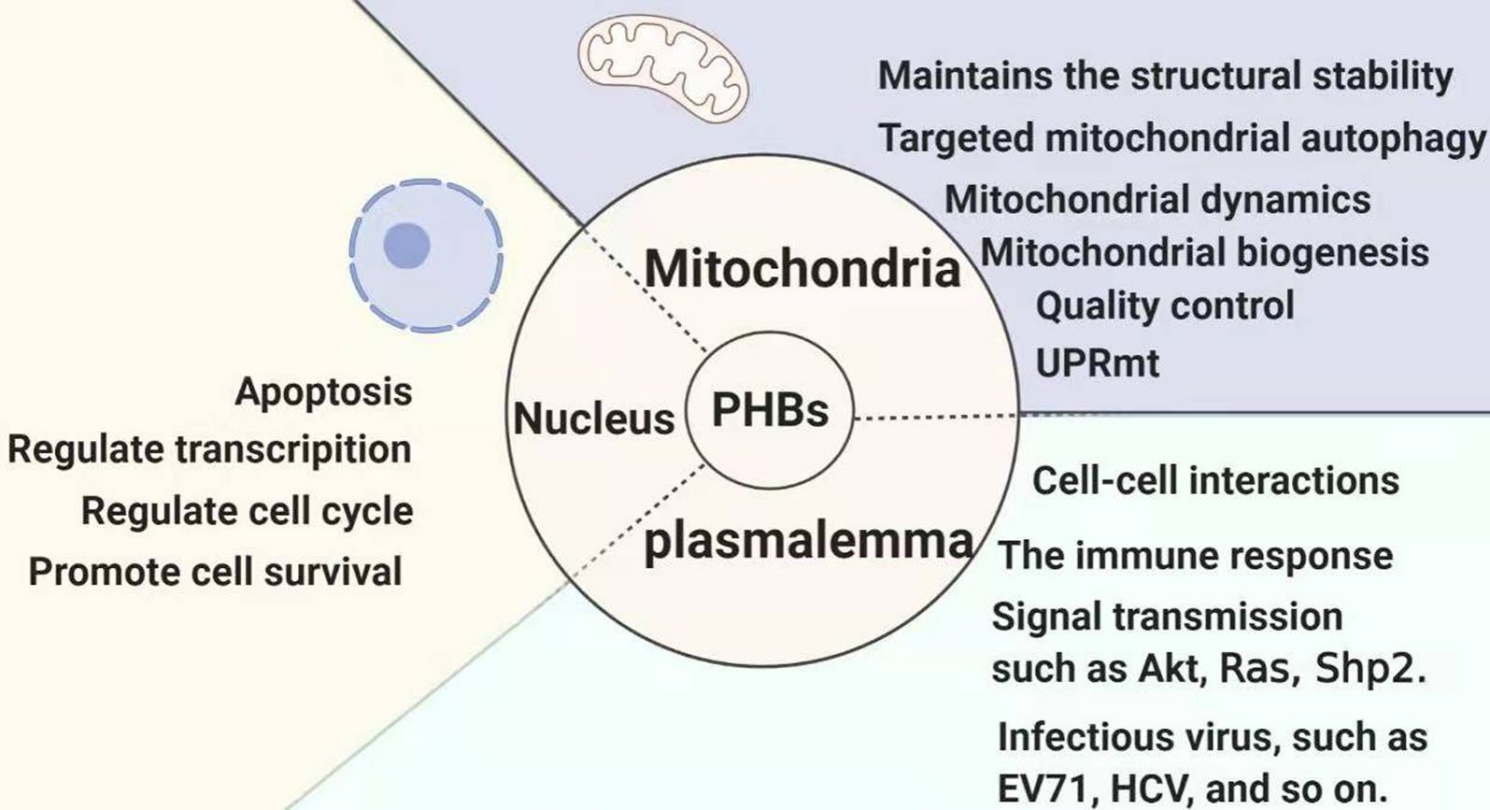
The subcellular location and function of PHB. PHB is widely distributed in both prokaryotic and eukaryotic cells, and its different subcellular location determines its different functions. In the cell nucleus, PHB binds to transcription factors to regulate gene transcription and translation, cell proliferation and metabolism, and cell growth cycle, affecting cell survival and autophagy. In the cytoplasm, PHB acts as a signal transduction molecule for molecules such as Akt, Ras and Shp2. It is an important molecule that affects cellular immune regulation, and it is also a helper for some viruses to invade cells, In the mitochondria with the most PHB distribution, PHB complex acts as the skeleton of mitochondria, maintains the functions of mitochondrial cristae and mitochondrial membranes, and participates in mitochondrial membrane division and fusion, mitochondrial quality control, non-coding protein balance, mitochondrial biogenesis and mitochondrial autophagy and other functions. These functions in mitochondria interact with each other, regulate and cooperate with each other to complete complex biochemical reactions in mitochondria

PHBs are located in the nucleus, cytoplasm, and mitochondria [13], and the location determines the functions of PHBs (Fig. 1). PHBs are rarely observed in the cytoplasm. Under normal physiological conditions, PHBs shuttle between the nucleus, cytoplasm and mitochondria through the nuclear pore complexes and act as signaling molecules between the nucleus and mitochondria. In melanocytes, Rab27a-PHB-MLPH complexes can control the pigmentation and hyperpigmentation of melanosome [14]. On the cell surface, such as B cells, platelets, and adipose tissue, PHB has a role in cell-to-cell signaling. In intestinal mucosal epithelial cells, PHB is not only the binding site of the capsular polysaccharide of Salmonella typhimurium Vi, but also the receptor of some viruses, such as hepatitis C virus, Chikungunya virus EV-A71 virus, and so on [15–18].

In mitochondria, PHBs mediate mitochondrial cristae stabilization, mitochondrial autophagy, stabilization of mitochondrial dynamics, mitochondrial biogenesis and quality control, and are involved in mitochondrial unfolded protein reactions. PHBs expressed on mitochondria can allow many viruses to enter the cell by interacting with the cell membrane. EV71 enterovirus specifically enters the neuron, while the membrane bound mitochondrial PHB1 associates with the virus replication complex and promotes virus replication. Besides, Roc-A treatment of EV71-infected neurons could significantly reduce virus production. However, the mechanism of the inhibitory effect of Roc-A on PHB in NSC-34 cells is not achieved by blocking the CRAF-MEK-ERK pathway but the impairment of mitochondrial integrity. PHBs located on the plasma membrane are the obligatory for HCV to infect hepatocytes. At present, it is believed that the C-terminus of PHB1 binds to the hepatitis C virus, which mediates the virus to enter the later stage of hepatocytes [19]. After the C-terminus of PHBs is knocked out, HCV are not able to infect hepatocytes. PHB1 is also involved in the entry of DENV-2 and chikungunya virus (CHIKV) [20], in which the complex of PHB1 and PHB2 is identified as the receptor of dengue virus into SH-SY5Y and CHME-3 cells, and binds to the HIV-1 glycoprotein and envelope protein of leukoplakia syndrome virus [19].

## The role of PHBs in mitochondria

**Fig. 2:**
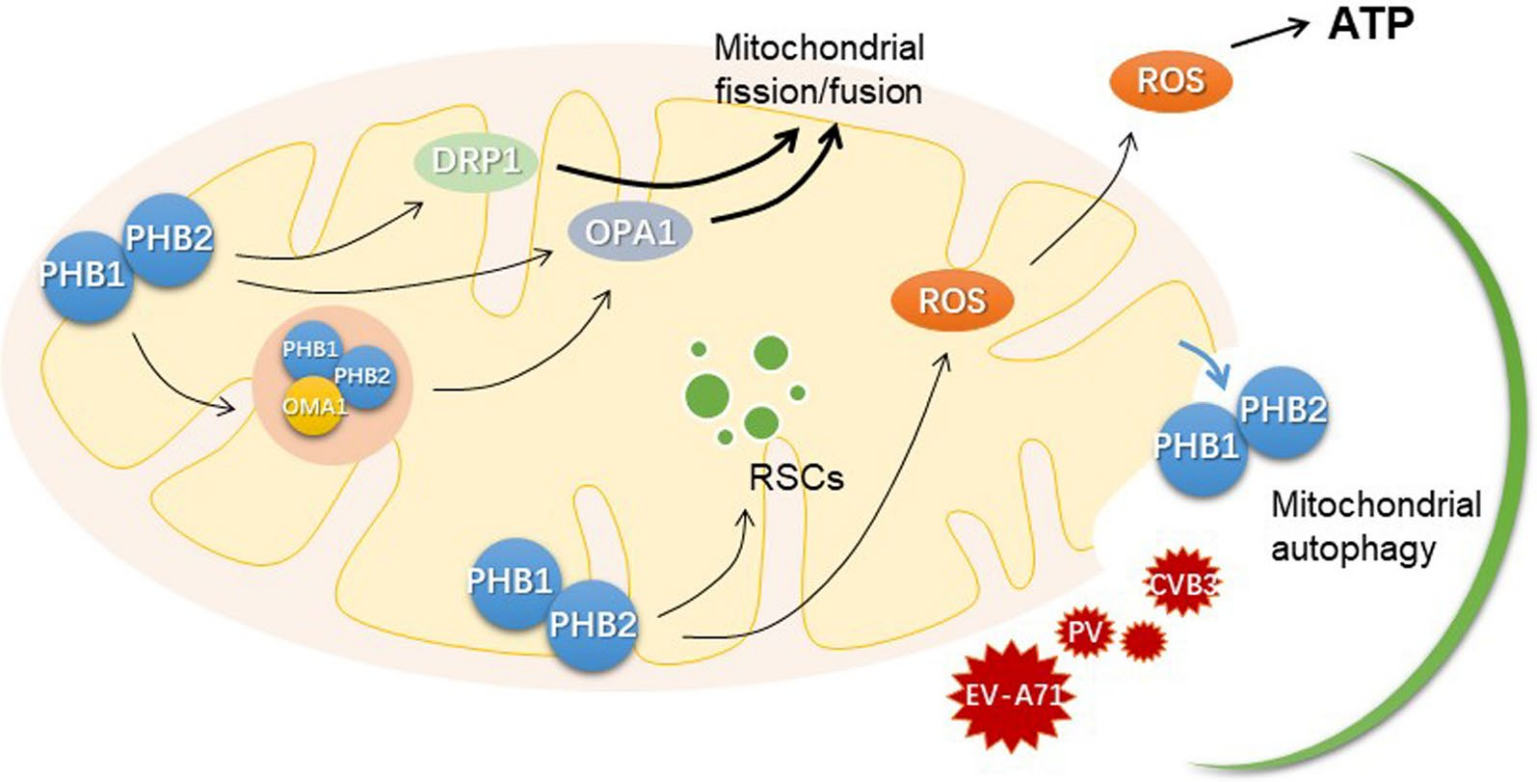
The function of PHBs in mitochondria. PHBs in mitochondria interact to maintain complex cellular functions. The PHBs complex, consisting of PHB1, PHB2 and OMA-1, affects mitochondrial dynamics by regulating the activity of SLP2 and the ratio of L-OPA to S-OPA on the mitochondrial membrane. PHBs also regulate mitochondrial dynamics by directly regulating molecules associated with mitochondrial division and fusion, such as DRP-1, MFN-2, OPA1, etc. Meanwhile, mitochondria are highly active centers of cellular material and energy, and PHBs regulate mitochondrial scintillation, mainly by regulating RSCs, which are often accompanied by the production of large amounts of metabolites and free radicals to provide energy to cells. For damaged, stressed and aged mitochondria, PHB2 on the inner mitochondrial membrane is exposed to the cytoplasm, recruiting autophagic receptors and regulating autophagy through classical and non-classical pathways of autophagy

PHBs are involved in a variety of mitochondrial functions, such as mitochondrial structural stability, mitochondrial dynamics, mitochondrial biogenesis and quality control, mitochondrial autophagy and the mitochondrial unfolded protein response (UPR^mt^), which suggests PHBs act as multiple functions in the physio pathological processes (Fig. 2).

### PHBs maintain the structural stability of mitochondria

The integrity and stability of mitochondrial structure are the basis for mitochondria to function. Mitochondria membranes contain the inner mitochondrial membrane, the outer mitochondrial membrane, and a small intermembrane space between those. PHBs maintain the cristae structure and mitochondrial integrity to provide the place for biochemistry. In the cells with high demand for ATP, PHB1 protein is highly expressed [21], while in different organelles of the same cell, mitochondria are also distributed in the areas where energy demand is strong, suggesting that the energy supply of mitochondria is related to the expression of PHB1 [22]. Accordingly, the transcription and translation of PHB1 in senescent cells are less than those in normal cells [23]. That PHBs can stabilize and promote the production of ATP has been confirmed [24, 25]. There are exceptions that occur. For example, without PHB1 expression in Hela cells, the structure and morphology of mitochondrial cristae showed swelling and roundness of mitochondria, shortening and decreasing normal mitochondria, and down-regulation of mitochondrial membrane potential, but the silence of PHB1 had limited effect on ATP [26]. Interestingly, changes of endoplasmic reticulum Ca^2+^ can induce the emergency response of the endoplasmic reticulum and affect the function of mitochondria. This leads to changes in energy metabolism and substances (such as the tricarboxylic acid cycle, mitochondrial oxidative respiratory chain, lipid metabolism, etc.) in the inner membrane of mitochondria [27]. Studies have shown that the unique structure of PHB2 enables the protein-rich mitochondrial inner membrane to form a protein-free region and can make PHB2 protein negatively charged to maintain the stability and function of mitochondria. After knockout of the PHB1 gene, the nematode embryo cannot survive, which may be connected with the abnormal ATP production [28]. This proves that it is vital to life.

### PHBs mediate mitochondrial dynamics

Mitochondria adapt to the energy and material needs of cells under stress by continuously dividing and fusing. When nutrients are scarce and large amounts of energy are required, mitochondrial fusion is activated to ensure the supply of ATP. When energy demand is high and material supply surges, mitochondrial fission is activated to reduce the supply of ATP while generating ROS. This change in mitochondrial self-adaptation depends on the need for cellular dynamics and is referred to as mitochondrial dynamics [23]. Ratio and configuration changes of L-OPA1 and S-OPA1 are crucial for the fusion and division of mitochondrial membranes. The correct assembly of PHBs complexes, m-AA protease, OMA1 protease, cardiolipin maturation and other substances that can directly/indirectly regulate the OPA1 protein play an important role in securing the mitochondria dynamics. At the same time, the metabolism of lipid components of mitochondria can change the fluidity of mitochondrial membranes [35, 43, 44]. L-OPA1 is a unique feature of mitochondrial division that is also associated with chemosensitivity. Knockdown of PHB1 prevents cisplatin-induced mitochondrial division and Oma1-mediated cleavage, as well as changes in OPA1 processing, suggesting that mitochondrial dynamics involved in PHBs play an important role in controlling tumor chemoresistance [46]. It has been shown that in the stress response of renal cells, Bif-1 and PHB2 jointly disrupt the structure of the PHB complex, induce hydrolytic inactivation of OPA1 protein, and participate in the decrease of mitochondrial dynamics, mitochondrial rupture and activation of apoptosis [45]. This suggests that PHBs can stabilize mitochondrial membrane division and fusion processes under stress. The ability to respond to cellular energy demand cues when demand increases is particularly important in nerve cells and muscle cells. Due to the non-renewability of nerve cells and cardiomyocytes, understanding of the mechanisms of PHBs in mitochondrial dynamics is an urgent problem [38, 39].

Glucose deprivation (GS) increase mitochondrial PHB1, leading to its dissociation from Dynamin-related protein 1 (DRP1), disrupting the mitochondrial membrane potential and triggering the apoptotic cascade [42]. DRP1 is an essential protein required for mitochondrial division, and the fusion of the mitochondrial outer membrane (MOM) depends on the functional interaction of mitogenic proteins (Mfn1 and Mfn2). When mitochondria divide, DRP1 is located in the cytoplasm and the mitochondrial outer membrane forms oligomers that wrap and divide the mitochondrial outer membrane [40]. DNM1-3 motor proteins play an auxiliary role in the division of mitochondria [41].

### PHBs act in mitochondrial biogenesis and quality control

In mitochondria, the transportation of materials and the production of energy needs to achieve dynamic balance to maintain the normal life of cells, which is called mitochondrial quality control [23]. PHBs deficiency reduces the formation of mitochondrial respiratory super complexes (RSCs), but does not change the abundance of individual respiratory complex subunits. The co-expression of PHB1 and PHB2 effectively rescues the damages caused by PHBs deficiency, indicating that the multimeric PHB complex functions as a functional unit. PHBs mainly modulate mitochondrial flashes by regulating the production of RSCs and ROS [47]. Studies have shown that in 3T3-L1 preadipocytes, mitochondrial dysfunction and down-regulation of adipogenesis occur after PHBs silencing [48, 49]. In heart cells, knocking out PHB2 can cause carnitine palmitoyl transferase 1b (CPT1b) to induce cardiac fatty acid metabolism disorders [50].

Also, in the adipose tissues of mice, PHB1 phosphorylated transgenic mice have different responses to insulin resistance than the fat cells of PHB1 overexpression mice, different genders and different parts of fat cells respond to PHB1 deletion/overexpression differentially and manifest as a difference in metabolic abnormalities [51]. Interestingly, fat cells that overexpress or silence PHB1 expression in female mice are not abnormal, which may be related to sex hormone differences under different genders. It is believed that the fat with PHB1 regulates the copy number of mitochondrial DNA through PGC-1α, TFAM, and other co-transcriptional regulators, and the overexpression of PHB1 leads to an increase in nuclear-mitochondrial communication, which may lead to changes in mitochondrial biology [22]. In ovarian cancer cells, there is a view that PHB2 also causes an increase in mitochondrial biogenesis, which may be related to the signaling pathways of SIRT3, OPA1, and PHB2 proteins in camp, but the mechanism of how PHB2 mediates biogenesis is still unclear.

### PHBs and autophagy

Autophagy is an important antiviral defense mechanism of cells, but some viruses have evolved strategies to use autophagy to promote self-replication. Many RNA viruses, such as poliovirus (PV), coxsackievirus B3 (CVB3), Japanese encephalitis virus (JEV) and hepatitis C virus (HCV), enterovirus A71 (EV-A71), COVID- 19 can induce autophagy and use autophagic vesicles to promote self-replication, and studies have shown that silencing the expression of PHB2 can increase the survival of cells infected by EV-A71 survival of cells infected with EV-A71 [37]. When the structure of mitochondria is damaged, ROS are released into the cytoplasm to damage other organelles, leading to cell death and mitochondrial autophagy that is a low hazard, selective measure like apoptosis [23]. Studies have found that although mitochondrial autophagy and apoptosis are two different physiological phenomena, they have common parts in regulatory factors and signal transduction pathways [29, 30]. When the outer mitochondrial membrane is ruptured, the PHB2 protein in the inner mitochondrial membrane is exposed to the cytoplasm and the autophagy-specific protein LC3-II is recruited by the PHB2 protein for direct binding with the intervention of the proteasome [31]. Through the PHB2-PARL-PGAM5-PINK1 axis, the autophagosome transports the damaged mitochondria into the lysosomes, the autolysosome is promoted by vacuolar ATPase, and the acidification and degradation of vesicles lead to complete autophagy [32, 33]. Binding of PHB2 to the C-terminus of the VP1 structural protein of EV-A71 is critical for the induction of autophagy and EV-A71 infectivity. Autophagy induced by the virus can enhance viral replication or evade immune surveillance. Beside PHB2, OPTN, DNP52, FUNDC1, BNIP3 and other autophagy mediators have also been found to mediate mitochondrial autophagy [34, 35]. Study suggests that blocking mitochondrial autophagy may have anti-tumor effects [36].

In animal cells, while depletion of PHB shortens the lifespan of wild-type animals, it enhances the lifespan of a large number of metabolically impaired mutants, including the target mutants of rapamycin complex 2 (TORC2), sgk-1 and Rict-1. Unfolded protein response induction upon PHB depletion extends lifespan of sgk-1 mutants through autophagy and probably modulation of lipid metabolism [37].

### PHBs and UPR^mt^

Mitochondrial unfolded protein response (UPR^mt^) is a cellular stress response related to mitochondria, refers to the way that abnormal proteins in mitochondria exceed the normal processing capacity by increasing the number of chaperone proteins, activating proteases to degrade proteins and processing abnormal proteins through mitochondrial autophagy [52]. The occurrence of unfolded proteins may be caused by the accumulation of unfolded proteins in mitochondria, abnormal mitochondrial biogenesis, abnormal mitochondrial autophagy mechanisms, etc. RICT-1 interacts with PHBs to regulate lifespan and is signaling with SGK-1 for the regulation of the UPR^mt^ [53]. The UPR^mt^, which transfers the protein from the mitochondria to the nucleus and reprocesses the protein, has been confirmed. Changes in mitochondrial function and MT-UPR activation are indispensable factors of pathology, including IBD and cancer [54].

## Potential values of PHBs in clinical applications

### PHBs and neurodegenerative diseases

In patient/animal models of neurodegenerative disease, researchers initially believed that defects in mitochondrial DNA contributed to the onset and progression of the disease. As research progressed, neurodegenerative diseases were found to be associated with mitochondrial dysfunction, mitotic efficiency and oxidative stress, accompanied by changes in cytosolic calcium homeostasis. The pathogenic mechanisms of some mitochondrial diseases have now been identified, such as common metabolic diseases such as Alzheimer's disease (AD) and Parkinson's disease [55, 56].

During the onset and progression of AD, a decrease in PHB2 levels and a specific reduction in the phosphorylation isoforms of PHB1 leads to mitochondrial imbalance, which is a driver of olfactory decline in the earliest symptoms of AD. The PHB complex is also a different driver of neurodegeneration at the olfactory level [57]. Amyloid beta peptide (Aβ)-induced dysfunction of the cholinergic system and mitochondria is a major risk factor for AD. Cinnamic acid (CA), an important polyphenolic antioxidant, may ameliorate Aβ-induced mitochondrial imbalance and oxidative stress by upregulating the expression of PHB1, PHB2, EAT3 and DRP1 genes [59]. At the same time, PHBs in different types of dementia can have opposite or the same performance, which is upregulated in mixed dementia (AD and Vascular dementia), but in the downregulation of frontotemporal degeneration and no changes in progressive nuclear ascending paralysis. The authors found that the decrease in the levels of PHB1 and PHB2 will cause the level of PHB1-PHB2 complex to decrease, and the PHB1-PHB2 complex modulates mitochondrial dynamics by reducing the generation of free radicals, and the complex assembly of mitochondrial respiration participates in the beneficial effects of neurons, and in the middle and late stages of AD, the expression of PHB1 subtypes phosphorylated at Thr258 and Y259 declines. These all indicate that PHBs are involved in the occurrence and development of AD and may become the early screening indicators of AD providing a theoretical basis. Additional, in a study on neurodegenerative diseases using mice as experimental models, it was found that PDD005 (a purine derivative drug that can interact with PHB1 and PHB2) can pass through the blood-brain barrier and increase the concentration in central nerve cells [31]. The expression level of PHBs in turn regulates the mitochondrial oxidative stress response, activates the Akt signaling pathway, inactivates the NF-kb signaling pathway, reduces the expression of interleukin-1β, and reduces the activation of astrocytes and microglia to reduce cognitive deficits [31]. In the substantia nigra of Parkinson’s disease and AD, PHBs are positively correlated with the stage of Parkinson’s disease severity, that is, the more advanced Parkinson’s disease, the lower the PHBs content, while in sporadic AD no changes in PHBs were found, and the level of PHBs in the frontal cortex increased. The authors also studied the content of ATP synthase in each tissue and found that the correlation between PHBs level and PHBs content is not single, which suggests different signal regulation of inhibition and ATP synthase is prohibited, but the reason for the normal levels of ATP synthase in the frontal cortex of pDLB is unclear. Over-expression of PHBs in the striatum blocked mitochondrial depolarization and motor dysfunction in PD mice, demonstrating that PHBs can affect neuroprotection and that increased PHBs levels protect dopaminergic neurons against mitochondrial ROS and cytotoxicity [58].

### PHBs and cancers

The molecularly targeted inhibition of PHBs is not only manifested in mitochondrial diseases, but also in the occurrence and development of tumors. PHBs are widely distributed in cells, and PHB1 exerts controversial impacts on cell proliferation in different cancers [60]. In addition, many studies support PHBs as a worse prognosis factor in tumors, such as breast cancer [61], gallbladder carcinoma [62], Wilms' tumor [63], bladder cancer [64], gastric carcinoma [65], and Nasal Extranodal Natural Killer/T Cell Lymphoma [66]. PHBs is the independent prognostic factor for worse prognosis in Esophageal Squamous Cell Carcinoma [67], pancreatic cancer [68] and squamous cell carcinoma of the lung [69]. In Wilms' tumor, urine PHBs ELISA is closed to the Wilms' tumor relapse [63]. In nasopharyngeal carcinoma [70] and liver cancer [2], PHB often be regarded as a great biomarker.

PHB participates in cancer cell proliferation, differentiation, migration, and invasion by regulating cell proliferation, autophagy, and expression of downstream receptors and through its own biological characteristics. In a variety of tumors, PHB has been found to participate in a variety of signaling pathways that affect cell survival, proliferation and metastasis, but there is a lack of specific functions of PHB in different subcellular locations. In breast cancer, the interaction of PHB1 with p53 and E2F can promote the apoptosis of breast cancer cells, thereby inhibiting the proliferation and metastasis of breast cancer. Evidence shows that in gallbladder cancer, overexpression of PHB1 promotes cell proliferation and invasion through the activation of the ERK pathway. It was found in our previous work that PHB1 upregulated by Long-palate, lung and nasal epithelium clone 1 (LPLUNC1) enhances the anti-tumor effects in nasopharyngeal carcinoma through counteract TRIM21-mediated ubiquitination to inhibit the NF-κB activity [71]. PHB1 activates the p53 pathway, promotes the up-regulation of p21CIP1, and down-regulates the regulatory proteins in the G0/G1 phase, thus achieving the effect of inhibiting the proliferation of liver cancer cells. Meanwhile, liver-specific Phb1 knockout (KO) mice develop hepatocellular carcinoma (HCC) spontaneously by regulating MAX, MNT and MATα1 [72]. Overexpressed SLP2 competed against E3 ubiquitin ligase SKP2 to bind with PHB1 and decreases the ubiquitination level of PHB1, causing gastric cancer progression, and the authors attributed to SLP-induced activation of the MAPK signaling pathway [73, 74].

The molecular targeted inhibitors of PHB1 include ERAP, xanthohumol, Fluorizoline [75], RocA (flavaglines/rocaglamide) [60], etc. Both of RocA and Fluorizoline are products from medicinal plants. It has been shown that RocA and Fluorizoline inhibit the activation of the Raf-1/ERK signaling cascade and suppress cancer cell growth and metastasis by binding PHB1. Fluorizoline is a new synthetic molecule that induces p53‐independent apoptosis by selectively targeting PHBs. NOXA, BIM and PUMA have been shown necessary for fluorizoline-induced apoptosis and the induction of NOXA and PUMA is dependent on PHB expression [75]. Fluorizoline induction of ATF3 and ATF4 to upregulation of NOXA transcription results in apoptosis [76]. Fluorizoline induces intracellular Ca2^+^ levels to rise swiftly and markedly that induces endoplasmic reticulum stress, and then promotes the phosphorylation of both initiation factor 2 (eIF2) and elongation factor 2 (eEF2), inhibiting protein synthesis and therefore inducing cancer cell death [77]. In colorectal carcinoma cells, phosphorylation of PHB1 at Thr258 blocked by FL3 (flavaglines) results in its nuclear translocation and binding to the Axin1 promoter, inhibiting Wnt/beta-catenin signaling via PHB1-dependent activation of Axin1 and even inactivating the APC (adenomatous polyposis coli) [78]. ERAP, a short synthetic peptide, has been reported to target PHB2 to antitumor, especially in Estrogen-dependent cancers. ERAP and xanthohumol induce cell cycle arrest by inhibiting the complex of PHB2-BIG3 and promote nuclear translocation of PHB2/REA complexes. Clearly, some scholars have found that the short peptide of ERAP and the natural product xanthohumol directly target PHB2 and prevent the occurrence and development of cancer [60]. In brief, PHBs have been becoming valuable biomarkers [79–82] and potential targets for novel drug development [63, 83–85].

### PHBs and infectious diseases

**Fig. 3:**
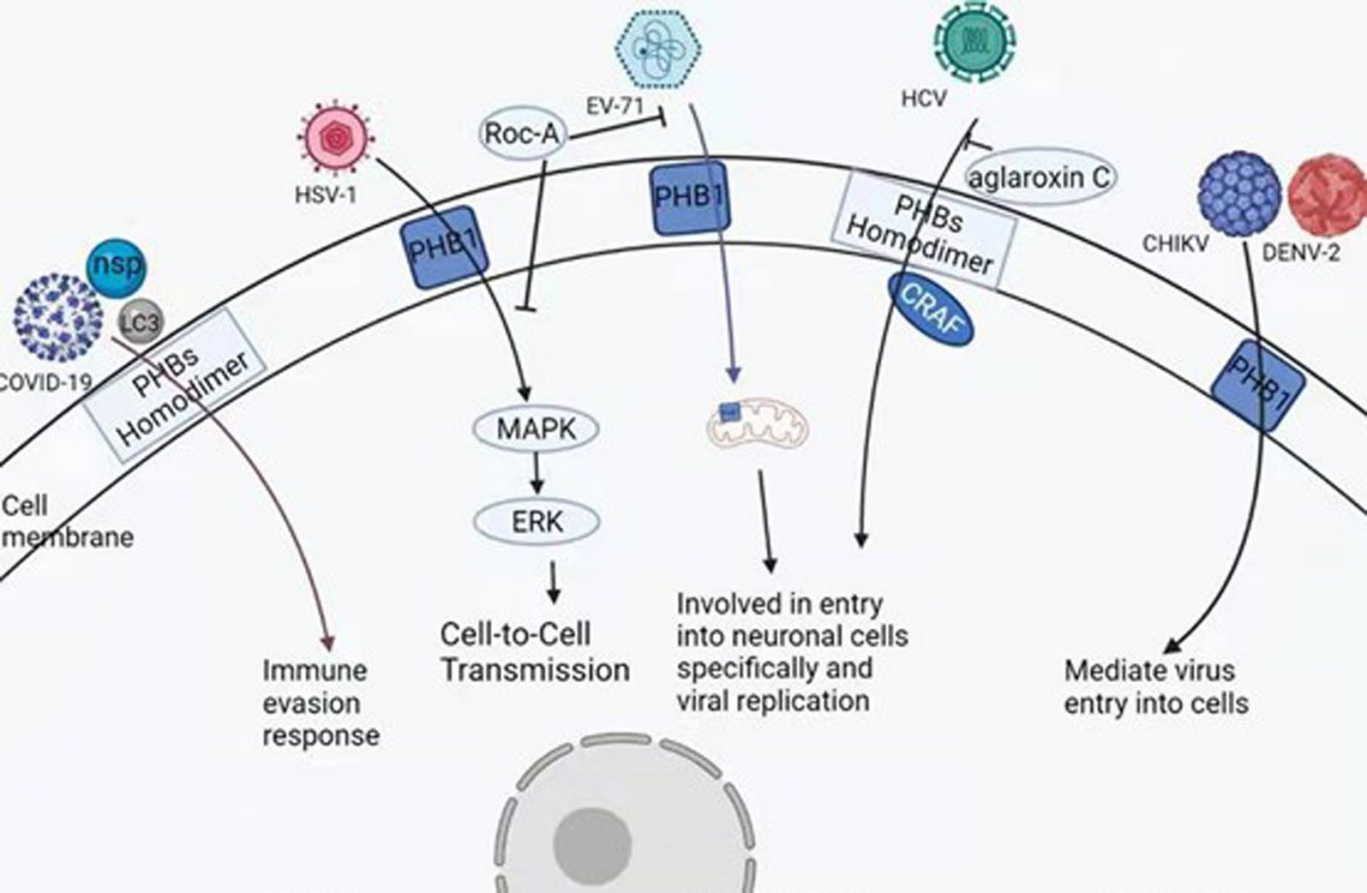
The PHB and viral infection. The PHB1 protein on the cell membrane assists HSV-1 to enter the cell and activates the MARK pathway, causing the virus to spread between cells. At the same time, PHBs on the cell membrane assist infection of EV-71 virus, CHIKV virus, DENV-2 virus, HCV virus and COVID-19 virus during cell infection. After COVID-19 infects cells, the virus exhibits immune escape ability. Even in the process of HCV infection, PHB knockout causes the HCV virus to lose its ability to infect, indicating that PHB is an indispensable molecule for HCV infection

As mentioned earlier, PHBs located at membrane act as the receptor of some viruses to assist the virus infection of cells. Endogenous PHB1 and PHB2 are necessary in the early stage of HCV infection. Further experiments proved that the PHB-CRAF interaction is the key to HCV entry, and the PHBs link H-Ras to CRAF-mediated signaling pathway. The Drugs targeting PHBs significantly reduced HCVcc, HCVpp, CHIKVpp, and dengue virus infection (Fig. 3) [19]. Natural and synthetic rocaglates prevent PHB1/2 from activating CRAF-mediated MEK-ERK pathway and thus prevent the virus into the cells. In EV-A71, the reduction of PHB1 can reduce virus replication and the incidence of critical illness. In COVID-19, PHB1 may mediate the host immune evasion response [19, 86, 87]. PHBs may bind to the NSP2 protein of the coronavirus to form an LC3-I-nsp2-PHB1/2 complex. Through the combination with a variety of non-structural proteins and ORF proteins, PHBs grant the ability of host immune evasion and virus replication, and natural flavonoids may be potential targeted drugs for the prevention of new coronary pneumonia because of their ability to inhibit eIF4A and PHBs [88].

With the deepening of clinical researches, PHB-related complications, such as metabolic syndromes, cardiovascular diseases, atherosclerosis, Parkinson's disease, and dyslipidemia in IBD patients have been discovered one after another [89]. It was found that PHB1 protein was downregulated in patients with IBD, and the specific knockout of PHB1 in intestinal epithelial cells leads to mitochondrial dysfunction of intestinal epithelial cells and Paneth cell dysfunction, inducing spontaneous ileitis in mice [90]. The specific knockout of PHB1 in Paneth cells can also induce ileitis. Mitochondrial antioxidants can improve the above-mentioned phenotypes caused by PHB1 deletion [90]. It is possible that PHB1 play a crucial role in the development and treatment of Crohn's disease. Flavonoids also take effective anti-inflammatory effects, especially in mouse models of Crohn’s disease [91]. These pharmacological effects are mediated by two types of molecular targets: the translation initiation factor eIF4A and the inhibitors 1 and 2 (PHB1 /2). It is a fact that a compound may affect PHBs, and other proteins undoubtedly increases the complexity of the drug's role in PHBs-related diseases. At the same time, PHBs under different modifications have different affinities with different compounds, which also pose a challenge for most scientific researchers to develop a PHBs-related drug with high efficiency and low toxicity.

Under hypoxia, NO directly acts on PHBs to nitrosylate PHBs and maintain the vitality of neurons, and thus NO can regulate the nitrosylation of PHBs and neuroprotective effect [12, 92].

## Conclusion

PHBs are widely distributed and present in different subcellular locations and organelles, and the different modified methods will affect its functions. The distribution in the mitochondria establishes mitochondrial stability, taking part in mitochondrial quality control, autophagy, and other functions. In fact, the functions of PHBs in the mitochondria are complementary to each other, and the stability of the mitochondria created for biological occurred in the mitochondria, mitochondrial protein folding reaction and mitochondrial autophagy are the most important functions of mitochondria self-renewal maintenance, mitochondria and mitochondrial dynamics and control of biological quality can further stabilize the structure of mitochondria to keep the mitochondria working properly. PHBs have clinical values in a variety of diseases. Some are involved in the occurrence and development of diseases, and others are used as a predictor or target of diseases. The corresponding clinical value has not been completely discovered at present, and there are different values and interpretations in different diseases. The clinical application value of PHBs should be continued to be discovered and improved and find the appropriate use and dosage of PHBs targeted drugs, which is still a problem to be solved in the clinical application of PHBs.

## Data Availability

The datasets used and/or analyzed during the current study are available from the corresponding author on reasonable request.
